# Visualization and quantitative analysis of extrachromosomal telomere-repeat DNA in individual human cells by Halo-FISH

**DOI:** 10.1093/nar/gkv091

**Published:** 2015-02-08

**Authors:** Martin Komosa, Heather Root, M. Stephen Meyn

**Affiliations:** 1 Genetics and Genome Biology Program, The Hospital for Sick Children, Toronto, Ontario, M5G 0A4, Canada; 2 Division of Clinical and Metabolic Genetics, The Hospital for Sick Children, Toronto, Ontario, M5G 1X8, Canada; 3 Department of Paediatrics, University of Toronto, Toronto, Ontario, M5S 1A8, Canada; 4 Department of Molecular Genetics, University of Toronto, Toronto, Ontario, M5S 1A8, Canada

## Abstract

Current methods for characterizing extrachromosomal nuclear DNA in mammalian cells do not permit single-cell analysis, are often semi-quantitative and frequently biased toward the detection of circular species. To overcome these limitations, we developed Halo-FISH to visualize and quantitatively analyze extrachromosomal DNA in single cells. We demonstrate Halo-FISH by using it to analyze extrachromosomal telomere-repeat (ECTR) in human cells that use the Alternative Lengthening of Telomeres (ALT) pathway(s) to maintain telomere lengths. We find that GM847 and VA13 ALT cells average ∼80 detectable G/C-strand ECTR DNA molecules/nucleus, while U2OS ALT cells average ∼18 molecules/nucleus. In comparison, human primary and telomerase-positive cells contain <5 ECTR DNA molecules/nucleus. ECTR DNA in ALT cells exhibit striking cell-to-cell variations in number (<20 to >300), range widely in length (<1 to >200 kb) and are composed of primarily G- or C-strand telomere-repeat DNA. Halo-FISH enables, for the first time, the simultaneous analysis of ECTR DNA and chromosomal telomeres in a single cell. We find that ECTR DNA comprises ∼15% of telomere-repeat DNA in GM847 and VA13 cells, but <4% in U2OS cells. In addition to its use in ALT cell analysis, Halo-FISH can facilitate the study of a wide variety of extrachromosomal DNA in mammalian cells.

## INTRODUCTION

Extrachromosomal nuclear DNA consists of DNA molecules that reside in the cell nucleus and are derived from genomic DNA, but are not covalently linked to chromosomes. Extrachromosomal nuclear DNA has been detected in all human tissues tested to date, raising the possibility that they may be involved in fundamental biological processes (1,2). These naturally occurring extrachromosomal DNA molecules range in length from <2 to >20 kb and are of diverse origin, including non-repetitive microDNAs as well as repetitive elements derived from satellite DNA and 5S ribosomal DNA (3,4).

Extrachromosomal DNA can also be generated under conditions of physiological or pathological stress (5). A classic example of this phenomenon is the extrachromosomal telomere-repeat (ECTR) DNA present in human immortalized and cancer cells that rely on the Alternative Lengthening of Telomeres (ALT) pathway(s) to maintain their telomere lengths (6,7). ALT is used by 10–15% of human tumors and is thought to be mediated by recombinational exchanges between DNA molecules containing telomere-sequence repeats (8,9). ECTR DNA in ALT cells can exist in single- or double-stranded forms, have linear or circular topology, and can form high molecular weight complexes (10–12). The exact origin and mechanism of ECTR DNA production in human ALT cells is currently not well understood, although the generation of circular ECTR DNA is dependent on several DNA repair proteins (13,14).

Currently, the primary tools used for ECTR DNA analysis are C-circle assay, electron microscopy and 2D agarose gel electrophoresis, techniques which are either technically demanding or semi-quantitative (10–12,15). Additionally, these cell-free techniques favor the study of circular DNA species. The design of the C-circle assay excludes linear ECTR DNA molecules from analysis, while with electron microscopy and 2D agarose gel electrophoresis, interpretation of ECTR DNA data typically excludes discussion of linear DNA molecules due to a potential for contamination by sheared linear chromosomal DNA. Importantly, these conventional methods for studying ECTR DNA cannot be used to obtain data from individual cells. This is a notable issue for ALT cell analysis, as a core characteristic of ALT cells is the marked cell-to-cell variability of their telomere-repeat DNA (16,17). While standard fluorescence *in situ* hybridization (FISH) techniques can be used to detect telomere-repeat DNA in individual cells, it is difficult to use these techniques to study ECTR DNA separately from chromosomal telomeres.

To overcome these technical limitations, we developed Halo-FISH, a FISH-based agarose gel technique, to visualize and quantitatively analyze extrachromosomal DNA molecules in individual cells. In the Halo-FISH assay, extrachromosomal DNA molecules are gently separated from chromosomes regardless of their topological conformation (linear or circular), under conditions that minimize shearing of chromosomal DNA. As a proof of principle, we demonstrate Halo-FISH by using the technique to provide detailed analyses of ECTR DNA molecules in individual human ALT and non-ALT cells. We detect few ECTR DNA molecules in primary and telomerase-positive cells, but markedly higher numbers in ALT cells. We report striking cell-to-cell variations in the number of ECTR DNA molecules in ALT cells, we quantify the wide distribution of ECTR DNA lengths in these cells and we provide evidence that the large majority of ALT ECTR DNA molecules are composed of primarily G- or C-strand telomere-repeat DNA. Furthermore, we report estimates, for the first time, of the fraction of the total telomere-repeat DNA content that is ECTR DNA in individual ALT cells. Lastly, we uncover ECTR DNA characteristics that are unique to specific ALT cell lines, suggesting that variant ALT mechanisms or genetic background differences between ALT cell lines can modulate the ECTR DNA phenotype.

The ability of Halo-FISH to uncover these novel ECTR DNA features in ALT cells demonstrates the technique's potential to facilitate the study of other extrachromosomal DNA species, including those that are present in the nuclei of healthy cells as well as those extrachromosomal DNA species that may arise in pathologic situations.

## MATERIALS AND METHODS

### Peptide nucleic acid probes and plasmid vectors

Peptide nucleic acid (PNA) probes used in this study are TelC-Rho (CCCTAACCCTAACCCTAA) human telomere PNA probe (PNA Bio Inc.), TelG-Cy5 (TTAGGGTTAGGGTTAGGG) human telomere PNA probe (Panagene) and CENPB-FAM (ATTCGTTGGAAACGGGA) human pan-centromere PNA probe (PNA Bio Inc.). Plasmid vectors pSXneo 135(T2AG3) with TTAGGG_135_ telomere DNA repeats and pSXneo 270(T2AG3) with TTAGGG_270_ telomere DNA repeats were obtain from Addgene.

### Cell culture

HT1080, HeLa1.2.11, GM00847 (GM847), WI-38 VA13/2RA (VA13) and U2OS cells were maintained in Dulbecco's Modified Eagle Medium supplemented with 10% fetal bovine serum (FBS). WI-38 cells were maintained in GIBCO Minimum Essential Medium Alpha Medium supplemented with 15% FBS. WI-38 cells were cultured from passage 22 to 26. All cell lines were cultured in a humidified 5% CO_2_ incubator at 37°C and are described in Supplementary Table S1.

### Cell processing and microscope slide preparation

Exponentially growing cells were harvested and resuspended in cold phosphate buffered saline (PBS), then 5–15 thousand cells were mixed in a 3:7 ratio with 1% low melting temperature agarose (Lonza) dissolved in PBS. Three-hundred microlitres of the resulting 0.7% agarose-cell mixture was pipetted onto each pre-warmed (45°C) TruBond 380 microscope slide (TruScientific) that had been previously coated with a thin layer of 1% low melting temperature agarose. A microscope slide coverslip (22 × 50 mm) was immediately placed on top to create a thin layer of the agarose-cell mixture, then the agarose was solidified by cooling to 4°C. The slides were placed in cold lysis solution (2.5M NaCl, 100 mM tetra-sodium EDTA, 10 mM Tris base, 1% N-Lauryl Sarcosine, 1% Triton X-100, pH 10.0) and coverslips were gently peeled off. The slides were left in lysis solution for 1 h at 4°C. After washing slides twice with cold ddH_2_O, slides were placed in 250 mM NaOH for 25 min. Immediately after, slides were serially dehydrated in 70, 90 and 100% ethanol and allowed to fully dry. Dried slides can be stored for up to 2 weeks at room temperature prior to probe hybridization without a detectable impact on subsequent hybridization or imaging.

### Fluorescence *in situ* hybridization

TelG-Cy5 PNA probe in hybridization buffer I (50% deionized formamide, 10% dextran sulfate, 1X SSC, 0.1 μg/ml herring sperm DNA) was pipetted onto prepared slides and spread over the thin layer of agarose gel with a microscope slide coverslip (24 × 60 mm). Slides were placed in a humidified chamber and hybridized at 37°C overnight. The next day, the slides were cooled in cold Wash Solution I (50% formamide, 2X SSC, pH 7.0) and coverslips were gently peeled off. Slides were washed three times with cold Wash Solution I, followed by three more washes with cold 0.1X SSC. Slides were serially dehydrated in 70, 90 and 100% ethanol and allowed to fully dry. These steps were repeated with the TelC-Rho and CENPB-FAM PNA probes together in hybridization buffer II (50% deionized formamide, 10% dextran sulfate, 1X SSC). Slides were counterstained with 0.1 μg/ml 4′, 6-diamidino-2-phenylindole (DAPI) for 5 min and mounted in SlowFade Antifade (Invitrogen) with microscope slide coverslips (24 × 60 mm).

### Fluorescence microscopy imaging

Cell nuclei were randomly selected for imaging and analysis, without regards to their Halo, by identifying the nuclear core of each deproteinized nucleus via their intense DAPI fluorescence. Each nuclear core was centered in the field of view and imaged by taking 40 z-stack single plane 12-bit grayscale widefield images at 0.2 μm intervals. All images were captured with a Zeiss Axioplan 2 microscope equipped with a Zeiss Plan-Apochromat 40x/0,95 Korr ∞/0,13–0,21 objective and a Hammamatsu Orca ER camera using Openlab software version 5.5.1 (PerkinElmer). z-stack images for DAPI, FAM, Rho and Cy5 fluorescent channels were captured for each deproteinized nucleus.

### Quantitative analysis

Individual sets of z-stack images for each deproteinized nucleus were subjected to iterative deconvolution and quantitative analysis using Volocity software version 6.0 (PerkinElmer). Three replicate experiments of 20 randomly selected nuclei from each cell line were processed for a total of 60 nuclei per cell line. The nuclear core of each deproteinized nucleus was defined by intense DAPI fluorescence. To convert telomere-repeat DNA fluorescent intensity measurements into DNA base-pair length estimates, a quantitative FISH calibration was performed as previously described (18). This involved seeding separate slides with plasmid DNA vectors containing inserts of 135 (810 bp) or 270 (1620 bp) telomere-sequence repeats and processing them in parallel with slides containing cell nuclei under study. The average PNA intensity of fluorescent foci associated with the plasmid DNA vector containing 135 (810 bp) telomere-sequence repeats was used to set a minimum threshold for detecting ECTR DNA molecules on slides run in parallel. Prism software version 5.00 (GraphPad) was used to perform T-tests to determine statistical significance.

## RESULTS

### Development of the Halo-FISH protocol to detect extrachromosomal DNA

**Figure 1.**
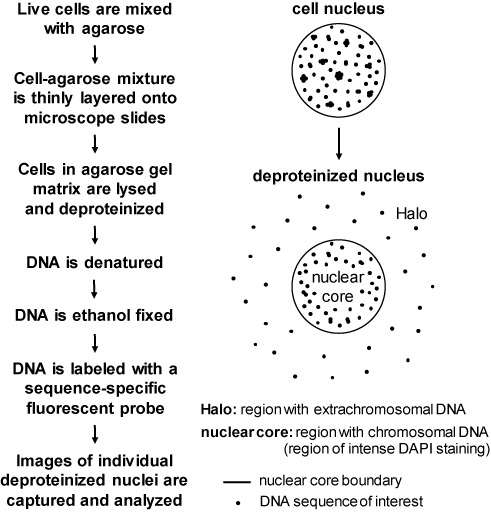
Overview of the Halo-FISH protocol. Live mammalian cells are harvested, mixed with agarose and thinly layered onto microscope slides. The cells trapped in the agarose gel matrix are lysed and deproteinized, then treated with NaOH to denature DNA and break up DNA aggregates. During this process, extrachromosomal DNA molecules diffuse out of the ‘nuclear core’ region and into the ‘Halo’ region of each deproteinized nucleus. The slides are subsequently ethanol dehydrated and fixed, followed by FISH with a fluorescently labeled sequence-specific probe to detect the extrachromosomal DNA of interest. Deproteinized nuclei are individually imaged and the captured image z-stacks are subjected to iterative deconvolution for quantitative analysis.

Halo-FISH is a FISH-based agarose gel technique that combines elements of the comet assay (19–23) and quantitative-FISH procedures (24) to detect and quantitatively analyze extrachromosomal nuclear DNA in individual mammalian cells (Figure 1). The procedure involves mixing as few as 200 live cells with low melting temperature agarose, then thinly layering this mixture onto microscope slides. Once the agarose has solidified, the cells trapped in the agarose gel matrix are lysed and deproteinized with a high salt and detergent buffer. The embedded deproteinized cell nuclei are then exposed to NaOH to denature their DNA, so as to break up any DNA aggregates and make the DNA accessible for subsequent hybridization with fluorescently labeled sequence-specific probes.

Despite deproteinization and DNA denaturation, size constraints prevent chromosomal DNA from diffusing significant distances through the agarose gel matrix. Consequently, chromosomal DNA from a single deproteinized cell nucleus is primarily contained within a ‘nuclear core’ region that can be clearly defined with the DNA counterstain DAPI. In contrast, following deproteinization and subsequent exposure to NaOH, individual extrachromosomal DNA molecules freely diffuse away from the nuclear core to form a ‘Halo’ of DNA molecules. Optimal denaturation conditions are chosen to allow for the diffusion of extrachromosomal DNA molecules away from chromosomes confined to the nuclear core (as visualized by intense DAPI staining), but to limit their diffusion distances within the Halo so as to not escape the field of view of the microscope objective when a deproteinized cell nucleus is centered for imaging.

After DNA diffusion, the agarose gel is dehydrated, thereby fixing the DNA in place. Standard FISH techniques are then used to detect the DNA of interest with a fluorescently labeled sequence-specific probe. z-stack images are captured for individual cell nuclei and are subjected to iterative deconvolution, followed by quantitative analyses of both the number and size of the extrachromosomal DNA molecules.

### Halo-FISH detects ECTR DNA molecules in human cells

In human cells, ECTR DNA molecules are composed of G-strand (TTAGGGn) and/or C-strand (AATCCCn) telomere-repeat DNA that can vary greatly in length (10). To detect the ECTR DNA, two fluorescently labeled PNA probes were used; one targeted for the G-strand and the other for the C-strand of telomere-repeat DNA. The high affinity and specificity of PNA probes make them suitable for quantitative analysis (25).

**Figure 2.**
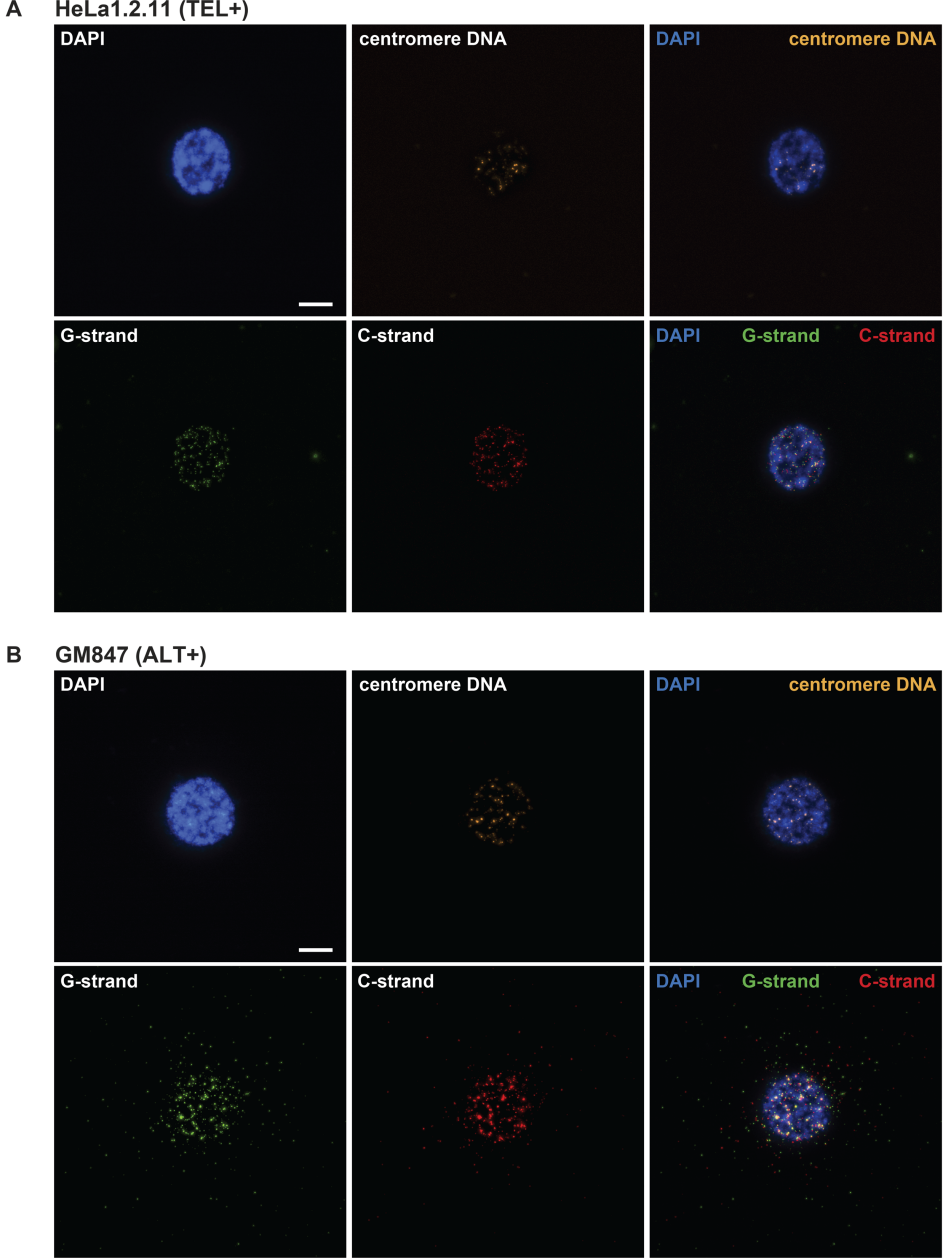
Representative images of human cells subjected to the Halo-FISH protocol. HeLa1.2.11 telomerase-positive cell (**A**) and GM847 ALT cell (**B**) are PNA probed for centromere DNA (orange), G-strand telomere-repeat DNA (green), C-strand telomere-repeat DNA (red) and counterstained with DAPI (blue). A set of 40 z-stack images were captured for each deproteinized nucleus at 0.2 μm intervals and were subjected to iterative deconvolution. TEL+: telomerase-positive, ALT+: ALT-positive. Scale bar, 10 μm.

**Figure 3.**
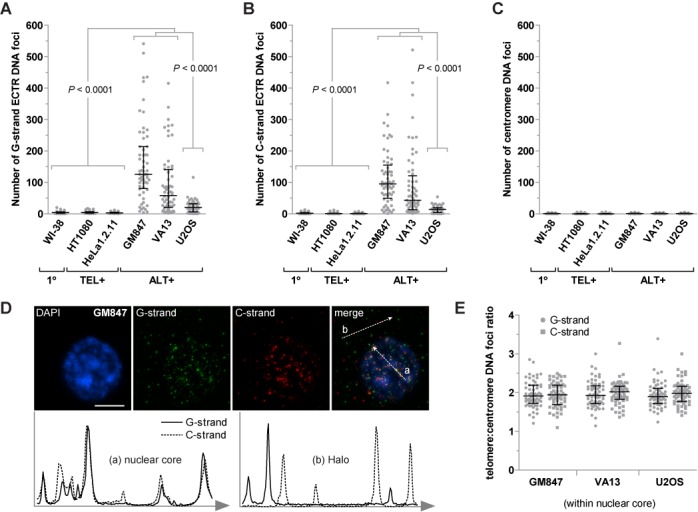
Analysis of the number of ECTR DNA molecules detected in human primary, telomerase-positive and ALT cells. The number of G-strand ECTR DNA foci per nucleus (**A**), the number of C-strand ECTR DNA foci per nucleus (**B**) and the number of centromere DNA foci per nucleus (**C**) located in the Halo of the deproteinized nuclei are plotted for WI-38 primary cell, HT1080 and HeLa1.2.11 telomerase-positive cells, and GM847, VA13 and U2OS ALT cells. (**D**) GM847 deproteinized nucleus counterstained with DAPI (blue) to visualize the nuclear core and probed for G-strand (green) and C-strand (red) telomere-repeat DNA. White arrows through the merged image indicate the direction of the intensity linescan plots. The intensity linescan plots show colocalization patterns between G- and C-strand telomere-repeat DNA signals within the (a) nuclear core and the (b) Halo of the deproteinized nucleus. Scale bar, 10μm. (**E**) G- or C-strand telomere to centromere DNA foci ratio per nucleus within the nuclear core of the deproteinized nuclei for GM847, VA13 and U2OS ALT cells. 1°: primary, TEL+: telomerase-positive, ALT+: ALT-positive. Median and error bars representing the interquartile range are displayed.

To demonstrate the utility of Halo-FISH, we used the technique to characterize ECTR DNA molecules in several human primary, telomerase-positive and ALT cell lines (Supplementary Table S1). WI-38 is a primary human fibroblast cell line that lacks detectable telomere maintenance mechanisms (26). The human fibrosarcoma cell line HT1080 and cervical carcinoma cell line HeLa1.2.11 both use telomerase for telomere maintenance (27,28). HeLa1.2.11, a clonal derivative of HeLa, has been reported to have telomeres that are substantially longer (15–30kb) than its parent (1–3kb) (28). However, the HeLa1.2.11 cells that we used in this study have telomeres that range from <3 to >20 kb in length (Supplementary Figure S2A and B). We analyzed 60 randomly selected nuclei per culture of exponentially growing cells and detect very few ECTR DNA molecules in the nuclei of WI-38, HT1080 and HeLa1.2.11 cells (Figures 2A and 3A and B). On average, these primary and telomerase-positive cells contain <5 detectable ECTR DNA molecules of either the G- or C-strand per nucleus, with the majority of the telomere-repeat DNA signals located within the nuclear core of the deproteinized nuclei (e.g. Figure 2A).

We next examined cells from GM847, VA13 and U2OS human ALT cell lines, which are commonly used in ALT studies, and find that they average 7- to 48-fold more ECTR DNA molecules per nucleus than primary and telomerase-positive cells (Figures 2B and 3A and B). GM847 is a SV40-immortalized human skin fibroblast cell line, while VA13 is a SV40-immortalized derivative of the WI-38 lung fibroblast cell line (29). We detect an average of 126 (G-strand)/95 (C-strand) and 59 (G-strand)/44 (C-strand) ECTR DNA molecules associated with the nuclei of GM847 and VA13 cells respectively (Figure 3A and B). Notably, the number of molecules per nucleus in both ALT cell lines varies widely from <20 to >300 molecules of each strand. U2OS osteosarcoma ALT cells (30) also display significantly more ECTR DNA molecules per nucleus compared to primary and telomerase-positive cells (Figure 3A and B). However, we find fewer ECTR molecules associated with the nuclei of U2OS cells than those of GM847 and VA13 cells, averaging only 21 (G-strand)/14 (C-strand) detectable ECTR DNA molecules per nucleus, with a range of 0 to >30 molecules of each strand (Figure 3A and B). We investigated the possibility that some of the molecules detected by Halo-FISH are the result of PNA probes hybridizing to telomere-repeat non-coding RNA (TERRA) molecules, which have been previously reported to be elevated in ALT cells (31). We find that pretreatment with DNAse I prior to probe hybridization completely eliminates detectable PNA fluorescent signals in the Halo-FISH assay, while pretreatment with RNAse A has no effect (Supplementary Figure S1), indicating that TERRA molecules do not generate false-positive ECTR DNA signals.

Interestingly, only 5.3, 6.8 and 8.2% colocalization events per nucleus are detected between the C- and G-strand ECTR DNA foci in GM847, VA13 and U2OS cells respectively. This suggests that the majority of the ECTR DNA molecules in ALT cells may be exclusively composed of G- or C-strand telomere-repeat DNA. In contrast, frequent colocalization is observed between the G- and C-strand foci within the nuclear core of deproteinized ALT nuclei (Figure 3D).

To verify that the telomere-repeat DNA signals detected in the Halo of the deproteinized nuclei are indeed non-chromosomal telomere-repeat DNA and that chromosomal loci remain primarily within the nuclear core, we examined the localization of centromere DNA, a repeat sequence that is also abundant in the nucleus. In contrast to telomere-repeat DNA foci (Figure 3A and B), we detect an average of <2 centromere DNA foci in the Halo of deproteinized ALT and non-ALT nuclei (Figure 3C), with the majority of centromere DNA signals located within the nuclear core (e.g. Figure 2A and B). The rare centromere DNA signals observed in the Halo of deproteinized nuclei may represent extrachromosomal centromere DNA, non-specific background staining or may be chromosomal in nature, as rare fibers protruding from the nuclear core into the Halo were apparent by examining nuclei stained with more sensitive SYBR Green and SYBR Gold DNA counterstains (data not shown). Furthermore, the average ratio of the number of G- or C-strand telomere-repeat DNA foci to centromere DNA foci within the nuclear core of deproteinized ALT nuclei is 2 (Figure 3E), as would be expected if chromosomal telomeres remained within the nuclear core while ECTR DNA molecules diffused out. Taken together, these results suggest that Halo-FISH separates ECTR DNA molecules from chromosomal telomeres, with most of the ECTR DNA molecules diffusing into the Halo of the deproteinized nucleus and the large majority of the chromosomal telomeres remaining in the nuclear core.

### ECTR DNA molecule length distributions in human ALT cells

To determine the length of each ECTR DNA molecule detected in human ALT cells, a quantitative FISH calibration was performed as previously described (18). Briefly, separate slides seeded with plasmid DNA vectors containing inserts of either 135 (810 bp) or 270 (1620 bp) telomere-sequence repeats were subjected to Halo-FISH, in parallel with slides containing cells under study, in order to convert telomere-repeat DNA fluorescent intensity measurements into DNA base-pair length estimates. As a proof of principle, we used this approach to measure the size of telomere-repeat DNA foci found within the nuclear core of deproteinized HeLa1.2.11 and HT1080 nuclei in the Halo-FISH assay (Supplementary Figure S2A). We then compared these measurements to telomere size estimates obtained by Southern blot analysis of telomere restriction fragments from the same two cell lines (Supplementary Figure S2B). We find that the telomere size distributions as determined by the Halo-FISH assay for these non-ALT cell lines parallel those obtained by Southern Blot analysis, with mean telomere sizes estimated by the two methods falling within ∼10% of each other. Thus, we conclude that our telomere-repeat DNA fluorescent intensity to DNA base-pair length conversions are sufficiently accurate to be used in estimating the length of DNA molecules that are longer than 1620 bp.

**Figure 4.**
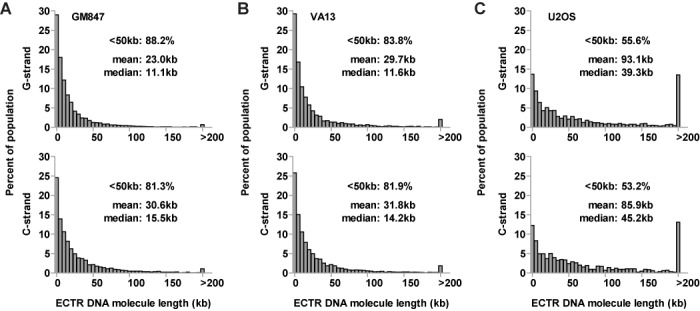
ECTR DNA lengths in human ALT cells. The length distributions of G- and C-strand ECTR DNA molecules are displayed for GM847 (**A**), VA13 (**B**) and U2OS (**C**) ALT cells. The percentage of foci <50 kb, as well as the mean and median lengths are indicated with each plot.

Using this fluorescence intensity:length in kb conversion approach, we find that the large majority of the ECTR DNA molecules detected in the nuclei of GM847 cells (G-strand: 88.2% and C-strand: 81.3%) and VA13 cells (G-strand: 83.8% and C-strand: 81.9%) are <50 kb in length (Figure 4A and B). The majority of the remaining molecules are between 50 to 200 kb, with <3% being >200 kb in length. The ECTR DNA molecules in U2OS cells are, on average, longer than in GM847 or VA13 cells. Almost half of the detectable ECTR DNA molecules (G-strand: 44.4% and C-strand: 46.8%) are >50 kb in length, while 13.5% (G-strand) and 13.1% (C-strand) of the molecules detected are >200 kb in length (Figure 4C). Notably, ECTR DNA lengths for all three ALT cell lines show markedly skewed distribution patterns, with their mean lengths being 2- to 2.5-fold greater than their respective median lengths (Figure 4).

### ECTR DNA content and cell cycle variation in human ALT cells

**Figure 5.**
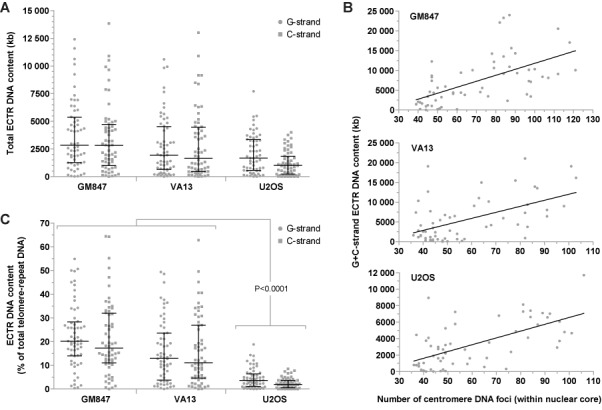
Analysis of ECTR DNA content in human ALT cells. (**A**) Total G- and C-strand ECTR DNA content per nucleus displayed for GM847, VA13 and U2OS ALT cells. (**B**) Correlation analysis, per nucleus, between the total G- and C-strand ECTR DNA content and the number of nuclear core-localized centromere DNA foci in GM847, VA13 and U2OS ALT cells. A linear regression slope with a confidence level of 95% is displayed with each plot. (**C**) Percentage of total telomere-repeat DNA, per nucleus, attributed to G- or C-strand ECTR DNA in GM847, VA13 and U2OS ALT cells. Median and error bars representing the interquartile range are displayed.

We find that, on average, GM847 cells contain the most and U2OS cells contain the least amount of ECTR DNA, as measured in total kb content (Figure 5A). Notably, asynchronously growing cells from the three ALT cell lines examined vary markedly in their total G- or C-strand ECTR DNA content, from <50 to >4000 kb per nucleus (Figure 5A). Therefore, we investigated the relationship between ECTR DNA production and cell cycle progression. As cells advance through the cell cycle, their chromosomes are replicated, thereby doubling the number of centromeres per nucleus. We find a positive correlation for all three ALT lines, per nucleus, between the total ECTR DNA content (G + C-strand) and the number of nuclear core-localized centromere DNA foci (Figure 5B).

To further explore the relationship between cell cycle progression and ECTR DNA production in ALT cells, we used nuclear core-localized centromere DNA foci counts to sort the deproteinized cell nuclei into G1-, S- and G2-enriched fractions. For the three ALT cell lines examined, G2-enriched nuclei average 2.7- to 4.9-fold more ECTR DNA molecules than G1-enriched nuclei (Supplementary Figure S3A). However, the median lengths of ECTR DNA molecules in G2-enriched nuclei average only to be 22.6% longer per nucleus than in G1-enriched nuclei (Supplementary Figure S3B).

Importantly, Halo-FISH separates ECTR DNA from chromosomal telomeres providing, for the first time, a way to measure the proportion of the total telomere-repeat DNA that is extrachromosomal. On average in GM847 and VA13 cells, G-strand ECTR DNA comprises 20.2 and 17.3% of the total G-strand telomere-repeat DNA content per nucleus respectively, while C-strand ECTR DNA represents 13.0 and 11.1% of the total C-strand telomere-repeat DNA content per nucleus respectively (Figure 5C). In contrast, the fractions of total G- and C-strand telomere-repeat DNA contents that are extrachromosomal in U2OS cells are significantly less. On average, ECTR DNA only makes up 3.6% (G-strand) and 2.0% (C-strand) of the total telomere-repeat DNA content per U2OS nucleus (Figure 5C). Notably, ECTR DNA comprises more than half of the total telomere-repeat DNA content in some GM847 and VA13 cells.

### G- and C-strand ECTR DNA biases in human ALT cells

**Figure 6.**
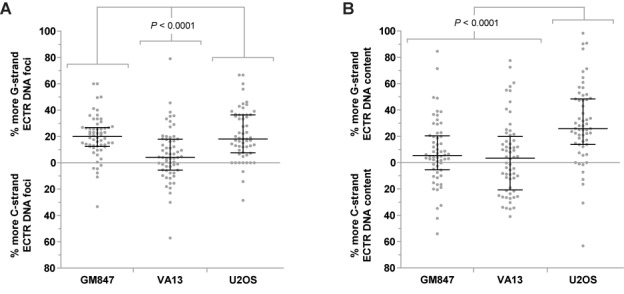
ECTR DNA strand biases in individual human ALT cells. (**A**) Biases in the number of G- or C-strand ECTR DNA molecules per nucleus are displayed for GM847, VA13 and U2OS ALT cells. (**B**) Biases in the total G- or C-strand ECTR DNA content per nucleus are displayed for GM847, VA13 and U2OS ALT cells. Median and error bars representing the interquartile range are displayed.

The ability to measure the number and lengths of both G- and C-strand ECTR DNA molecules in individual human ALT cells allows for the investigation into strand bias. Interestingly, GM847 and U2OS cells display an overall bias toward the production of G-strand ECTR DNA molecules. Ninety percent of GM847 nuclei contain more detectable G-strand ECTR DNA molecules with an average of 19.9% more G-strand molecules per nucleus, while 82.3% of U2OS nuclei have more G-strand ECTR DNA molecules with an average of 18.0% more G-strand molecules per nucleus (Figure 6A). In contrast, VA13 cells are only marginally biased in the generation of G-strand DNA molecules as only 61.7% of nuclei contain more detectable G-strand ECTR DNA molecules with only an average of 4.2% more G-strand molecules per nucleus (Figure 6A).

However, when total G-strand ECTR DNA content is compared to the total C-strand content, only U2OS cells display a very strong bias toward the G-strand. A total of 84.5% of U2OS nuclei have more G-strand ECTR DNA content with an average of 25.8% more G-strand ECTR DNA per nucleus (Figure 6B). In comparison, only 68.3 and 51.7% of GM847 and VA13 nuclei respectively have a G-strand ECTR DNA content bias, with an average of only 5.3 and 3.4% more G-strand ECTR DNA content per nucleus respectively (Figure 6B). We did not observe significant strand biases in telomere-repeat DNA foci number or content located in the nuclear core of the deproteinized nuclei for the three ALT cell lines examined (Supplementary Figure S4A and B).

## DISCUSSION

In this study, we present a novel strategy, Halo-FISH, to detect and quantitatively analyze extrachromosomal nuclear DNA in individual mammalian cells. This FISH-based agarose gel technique takes advantage of size-related differences in diffusion rates to gently separate extrachromosomal DNA molecules away from chromosomes. Following deproteinization of the cell nucleus and subsequent DNA denaturation, the majority of the extrachromosomal DNA molecules migrate out of the central nuclear core, which contains chromosomal DNA, and into the surrounding Halo. Those extrachromosomal DNA molecules of interest are visualized using a fluorescently labeled sequence-specific probe, then the number and size of the molecules are quantitatively analyzed. Halo-FISH is not technically laborious and complete datasets can be obtained with as few as 200 cells, which is particularly advantageous in studies that involve human stem and progenitor cells where cell yields are typically low. Importantly, Halo-FISH enables the study of extrachromosomal DNA on a single-cell basis and facilitates the examination of all detectable extrachromosomal DNA molecules of interest, regardless of their topological conformation (linear or circular).

The presence of ECTR DNA in human ALT cells is well established (6,7,10–12). Here we show that Halo-FISH detects individual ECTR DNA molecules in ALT cells and allows for their quantitative analysis. Several lines of evidence indicate that the telomere-repeat fluorescent signals detected in the Halo surrounding the nuclear core of deproteinized ALT nuclei represent ECTR DNA molecules. First, as expected, we detect significantly more ECTR DNA molecules in ALT cells compared to primary and telomerase-positive cells (Figure 3A and B). The majority of the molecules that we detect in GM847 and VA13 ALT cells are <50 kb in length, with a distribution that shows marked positive skewing (Figure 4A and B). This is in agreement with a previous report in which electron microscopy techniques were used to estimate the lengths of circular ECTR DNA molecules in GM847 and VA13 cells, ranging from 0.7 to 56.8 kb and showing positively-skewed length distributions similar to what we report (10). Second, we find very few detectable ECTR DNA molecules in primary and telomerase-positive cells, including HeLa1.2.11, which has relatively long telomeres that range up to 20 kb and more in length (Figure 3A and B, and Supplementary Figure S2). This indicates that in the Halo-FISH assay, shearing of chromosomal DNA does not occur frequently and that sheared chromosomal telomere-repeat DNA does not readily contribute to the ECTR DNA molecule populations that we report in ALT cells. This is further supported by the finding that other abundant repetitive chromosomal loci, such as centromere DNA, are rarely observed in the Halo of deproteinized ALT nuclei (Figure 3C). Lastly, pretreatment with DNase I completely eliminates PNA fluorescent signals while RNase A has no effect (Supplementary Figure S1), indicating that the telomere-repeat containing molecules described in this study are composed of DNA and do not represent TERRA molecules.

The Halo-FISH protocol provides, for the first time, a way to quantitatively analyze both ECTR DNA and chromosomal telomeres simultaneously in the same cell. The following observations indicate that Halo-FISH is effective in spatially separating ECTR DNA molecules from chromosomal telomere-repeat DNA. First, similar to previous reports (6,7), we detect very few ECTR DNA molecules in primary and telomerase-positive cells in the Halo-FISH assay (Figure 3A and B). Second, almost all detectable centromere DNA foci lie within the nuclear core of the deproteinized nuclei of primary, telomerase-positive and ALT cells (Figures 2 and 3C). Together, these two observations indicated that in the Halo-FISH assay, chromosomal DNA, including telomeres, does not readily diffuse into the Halo of the deproteinized nuclei where they might generate false-positive ECTR DNA signals. Third, on average, there are twice as many telomere-repeat DNA foci as centromere DNA foci within the nuclear core of deproteinized ALT nuclei (Figure 3E). This finding indicates that the majority of the ECTR DNA molecules have diffused out of the nuclear core of each deproteinized nucleus and the remaining telomere-repeat DNA foci that localize to the nuclear core likely represent chromosomal telomeres.

By using Halo-FISH to estimate the fraction of the total telomere-repeat DNA content that is extrachromosomal, we find that ECTR DNA can significantly contribute to the overall amount of telomere-repeat DNA within an ALT cell. For instance, ECTR DNA represents at least one quarter of the total telomere-repeat DNA content within the nuclei of ∼25% of the GM847 and VA13 asynchronous cell populations (Figure 5C). Additionally in certain ALT cells, we find that there is more ECTR DNA than there is chromosomal telomere-repeat DNA located in the nuclear core. Since the amount of ECTR DNA within an ALT cell is highly variable, measured differences in total telomere-repeat DNA content in two populations of ALT cells could be exclusively due to a change in ECTR DNA content, a change in chromosomal telomere length or a combination of both. Halo-FISH can distinguish between these possibilities in interphase cells, while standard FISH techniques are unable to. Conventional quantitative-FISH is typically used to measure telomere length strictly in metaphase cells, while Flow-FISH is used to monitor telomere length in asynchronous populations. However, caution should be taken when using Flow-FISH to estimate telomere lengths in ALT cell populations as this technique only measures the total amount of telomere-repeat DNA in a cell, which includes both telomeres and ECTR DNA.

Colocalization between G- and C-strand telomere-repeat DNA signals within the nuclear core of deproteinized ALT nuclei is quite common in the Halo-FISH assay (Figure 3D). This observation is consistent with the idea that these colocalized foci in the nuclear core represent the G- and C-strands of chromosomal telomeres, which are spatially confined due to the restricted movements of their parent chromosome within the agarose gel matrix. In contrast, a common feature of the three ALT cell lines examined in this study is that the G- and C-strand ECTR DNA foci located in the Halo of deproteinized ALT nuclei rarely colocalize. This finding leads to three key conclusions. First, the strong deproteinizing and denaturation conditions in the Halo-FISH assay are sufficient to separate complementary G- and C-strands of ECTR DNA. Second, the large majority of telomere-repeat DNA fluorescent signals detected in the Halo of deproteinized ALT nuclei are individual ECTR DNA molecules. The alternative is that most ECTR DNA foci in the Halo consist of two or more DNA molecules that independently diffused through the agarose matrix but ended up at the same final location. However, this is highly unlikely as at least 50% of such ECTR DNA foci would be expected, by chance, to harbor both G- and C-strand molecules and would be detected as colocalization events, contrary to our experimental results. Third, this infrequent colocalization between the G- and C-strand ECTR DNA foci implies that the majority of the ECTR DNA molecules generated in ALT cells are composed of primarily G-strand or primarily C-strand telomere-repeat DNA. The latter also suggests that the ECTR DNA molecules are generated using telomere-repeat DNA templates that are also composed of primarily G- or C-strand, and that once an ECTR DNA molecule is produced, it does not readily undergo random ligation or catenation events that would covalently link it with other ECTR DNA molecules found in the nucleus. As to the few G- and C-strand ECTR DNA foci that do colocalize, they may represent either a single molecule containing both G- and C-strand sequences, two purely G- and C-strand molecules that have randomly diffused within the vicinity of each other or G- and C-strand ECTR DNA molecules that are topologically linked (e.g. supercoiled double-strand circular ECTR DNA).

It is currently unknown whether ALT is one distinctive pathway or describes multiple telomerase-independent mechanisms of telomere maintenance (32). Although ECTR DNA has been found in every ALT cell line tested to date, the Halo-FISH assay detects significant differences in ECTR DNA between ALT cell lines. Our observations are consistent with the concept of multiple ALT mechanisms but may also be the result of differences in genetic background between ALT cell lines. In this regard, we find that both GM847 and VA13 ALT cells contain significantly more ECTR DNA molecules than U2OS ALT cells, with a very broad range of molecules per nucleus (Figure 3A and B). In addition, GM847 cells, but not VA13 cells, display an overall bias toward slightly shorter, but more, G-strand ECTR DNA molecules than C-strand molecules (Figures 4 and 6A). Furthermore, U2OS cells differ from both GM847 and VA13 cells, in that they contain significantly fewer, but longer, ECTR DNA molecules (Figures 3A and B, and 4). U2OS cells also have an excess of G-strand ECTR DNA content, a bias that is not strongly observed in either GM847 or VA13 cells (Figure 6B). These ECTR DNA strand biases that we report are unlikely to be the result of a PNA signal detection issue as we do not find significant strand biases in telomere-repeat DNA foci number or content located in the nuclear core of the deproteinized nuclei for the three ALT cell lines examined (Supplementary Figure S4). Rather, they are likely the result of intrinsic biological differences in the production and/or loss of ECTR DNA molecules between the three ALT cell lines.

The marked variability in both the number of ECTR DNA molecules and ECTR DNA content in the three ALT lines examined in this study led us to explore the effects of cell cycle progression on ECTR DNA. Halo-FISH was used to measure the number and lengths of ECTR DNA molecules, as well as to determine the number of nuclear core-localized centromere DNA foci for each deproteinized ALT nucleus. For the three ALT cell lines examined, we find that ECTR DNA content increases as ALT cells progress through the cell cycle (Figure 5B). This increase in ECTR DNA content is primarily a result of ALT generating new ECTR DNA molecules rather than elongating existing ones, as we find that the average numbers of ECTR DNA molecules in G2-enriched ALT nuclei are 2.7- to 4.9-fold greater than those of G1-enriched nuclei (Supplementary Figure S3A), while the median lengths of G2-enriched ECTR DNA molecules average only to be 22.6% longer per nucleus than their G1-enriched counterparts (Supplementary Figure S3B). New ECTR DNA molecules may be created using chromosomal telomeres as templates or by copying existing ECTR DNA molecules. Together, these observations imply that ECTR DNA molecules substantially increase in number but not in size as ALT cells progress through the cell cycle, ECTR DNA replication does not solely occur by semi-conservative replication and that many ECTR DNA molecules are lost upon the completion of mitosis. In addition, the marked cell-to-cell variability in the numbers of G- and C-strand ECTR DNA molecules suggests that replication of G- and C-strand ECTR DNA is not coordinated in ALT cells (Figure 6A).

A common feature of all human ALT cell lines is the presence of ALT-associated promyelocytic leukemia protein (PML) nuclear bodies (APBs) (33). Consistent with the idea that APBs function as platforms for ALT activity (32), previous reports indicate that ECTR DNA localizes to APBs (34). Using conventional immuno-FISH, we can detect intensely fluorescent telomere-repeat DNA foci that localize to APBs in cells from the three ALT cell lines used in this study (data not shown). However, these very intense telomere-repeat DNA foci are rarely observed in the nuclear core of deproteinized ALT nuclei in the Halo-FISH assay. Additionally, the number telomere-repeat DNA foci averaged twice the number of centromere DNA foci within the nuclear core of deproteinized ALT nuclei in the Halo-FISH assay (Figure 3E). Taken together, these observations strongly suggest that the large majority of the ECTR DNA molecules within APBs are not topologically linked, as ECTR DNA molecules are released from APBs under the strong deproteinizing and denaturation conditions in the Halo-FISH assay.

In this study, we set the minimum threshold used for detecting ECTR DNA molecules to be the average PNA intensity of fluorescent foci associated with plasmid DNA vectors containing 135 (810 bp) hexameric telomere repeats. However, this limit of detection could be reduced further if lower signal-to-background noise ratios could be tolerated. For example, the performance of our current imaging system suggests that we could detect individual molecules containing as few as 57 (342 bp) hexameric telomere repeats if we were willing to accept a signal-to-noise ratio of 2:1.

While we have used Halo-FISH to extensively study ECTR DNA that is common to human ALT cells, Halo-FISH can also facilitate investigations of ECTR DNA in human non-ALT cells when normal telomere structure and maintenance is perturbed. Examples include cultured human non-ALT cells that generate circular ECTR DNA as a result of modifications to or loss of the telomere shelterin component TRF2 (35), the telomere replication helicase RTEL (36), the DNA repair protein BRCA2 (37) and the ASF1 histone chaperone (38). Additionally, Halo-FISH can be used to assess ECTR DNA reported to be associated with the chromosome instability syndromes ataxia-telangiectasia and Fanconi anemia (39–41).

ECTR DNA is only one of many species of extrachromosomal mammalian DNA, some of which are found in healthy cells from normal tissues. These extrachromosomal DNA species can be restricted to specific cell types such as the T- and B-excision circles generated during V(D)J recombination in lymphocytes (42), while others, including non-repetitive microDNAs and repetitive elements derived from satellite DNA and 5S ribosomal DNA, can be found in healthy cells from multiple organs (3,4). On the other hand, certain extrachromosomal DNA species are associated with disease. For instance, the nuclei of malignant cells can accumulate disease-specific extrachromosomal DNA such as small *c-myc* and *mdr1*-containing episomes, as well as double-minute chromosomes that harbor amplified oncogenes (5). Additionally, cells from patients with genome instability disorders, such as Immunodeficiency, Centromere instability and Facial anomalies (ICF) syndrome and several of the trinucleotide repeat expansion diseases, may generate repetitive extrachromosomal DNA that do not originate from telomere-repeat sequences (43,44). While we developed Halo-FISH as a technique to quantitatively visualize ECTR DNA in human cells, it can be adapted to study many of these other extrachromosomal mammalian DNA species and aid multiple investigations into the roles that extrachromosomal DNA plays in health and in disease.

## SUPPLEMENTARY DATA

Supplementary Data are available at NAR Online.

SUPPLEMENTARY DATA
