# Supplementary material for: Visualization and quantitative analysis of extrachromosomal telomere-repeat DNA in individual human cells by Halo-FISH
Source: Nucleic Acids Res. 2015 Feb 8;43(4):2152–63. doi: 10.1093/nar/gkv091 (PMC4344523; doi:10.1093/nar/gkv091)
Supplement: SUPPLEMENTARY DATA [file supp_43_4_2152__index.html]

Visualization and quantitative analysis of extrachromosomal telomere-repeat DNA in individual human cells by Halo-FISH — Visualization and quantitative analysis of extrachromosomal telomere-repeat DNA in individual human cells by Halo-FISH — SUPPLEMENTARY DATA 

# Visualization and quantitative analysis of extrachromosomal telomere-repeat DNA in individual human cells by Halo-FISH

## SUPPLEMENTARY DATA

**Files in this Data Supplement:**

- SUPPLEMENTARY DATA
