# Supplementary material for: Visualization and quantitative analysis of extrachromosomal telomere-repeat DNA in individual human cells by Halo-FISH
Source: Nucleic Acids Res. 2015 Feb 8;43(4):2152–63. doi: 10.1093/nar/gkv091 (PMC4344523; doi:10.1093/nar/gkv091)
Supplement: SUPPLEMENTARY DATA [file supp_gkv091_nar-02948-x-2014-File008.pdf]

Supplementary Information

Visualization and quantitative analysis of extrachromosomal telomere-repeat DNA in individual human cells by Halo-FISH

Martin Komosa[1], Heather Root[1] and M. Stephen Meyn[1,2,3,4,*]

[1]Genetics and Genome Biology Program, The Hospital for Sick Children, Toronto, Ontario, M5G 0A4, Canada
[2]Division of Clinical and Metabolic Genetics, The Hospital for Sick Children, Toronto, Ontario, M5G 1X8, Canada
[3]Department of Paediatrics, University of Toronto, Toronto, Ontario, M5S 1A8, Canada
[4]Department of Molecular Genetics, University of Toronto, Toronto, Ontario, M5S 1A8, Canada

*To whom correspondence should be addressed. Tel: +1 416 813 8485; Fax: +1 416 813 4931; Email: stephen.meyn@sickkids.ca

Supplementary Table S1. Human cell line characterization

Cell line	TMM	Cell type	Reference
WI-38	None	Fibroblast (primary)	26
HT1080	TEL+	Fibrosarcoma	27
HeLa1.2.11	TEL+	Cervical carcinoma	28
GM00847 (GM847)	ALT+	Fibroblast (SV40)	29
WI-38 VA13/2RA (VA13)	ALT+	Fibroblast (SV40)	29
U2OS	ALT+	Osteosarcoma	30

TMM: Telomere Maintenance Mechanism; **ALT+**: Alternative Lengthening of Telomeres-positive; **TEL+**: Telomerase-positive; **SV40**: immortalization with Simian Virus 40 genes

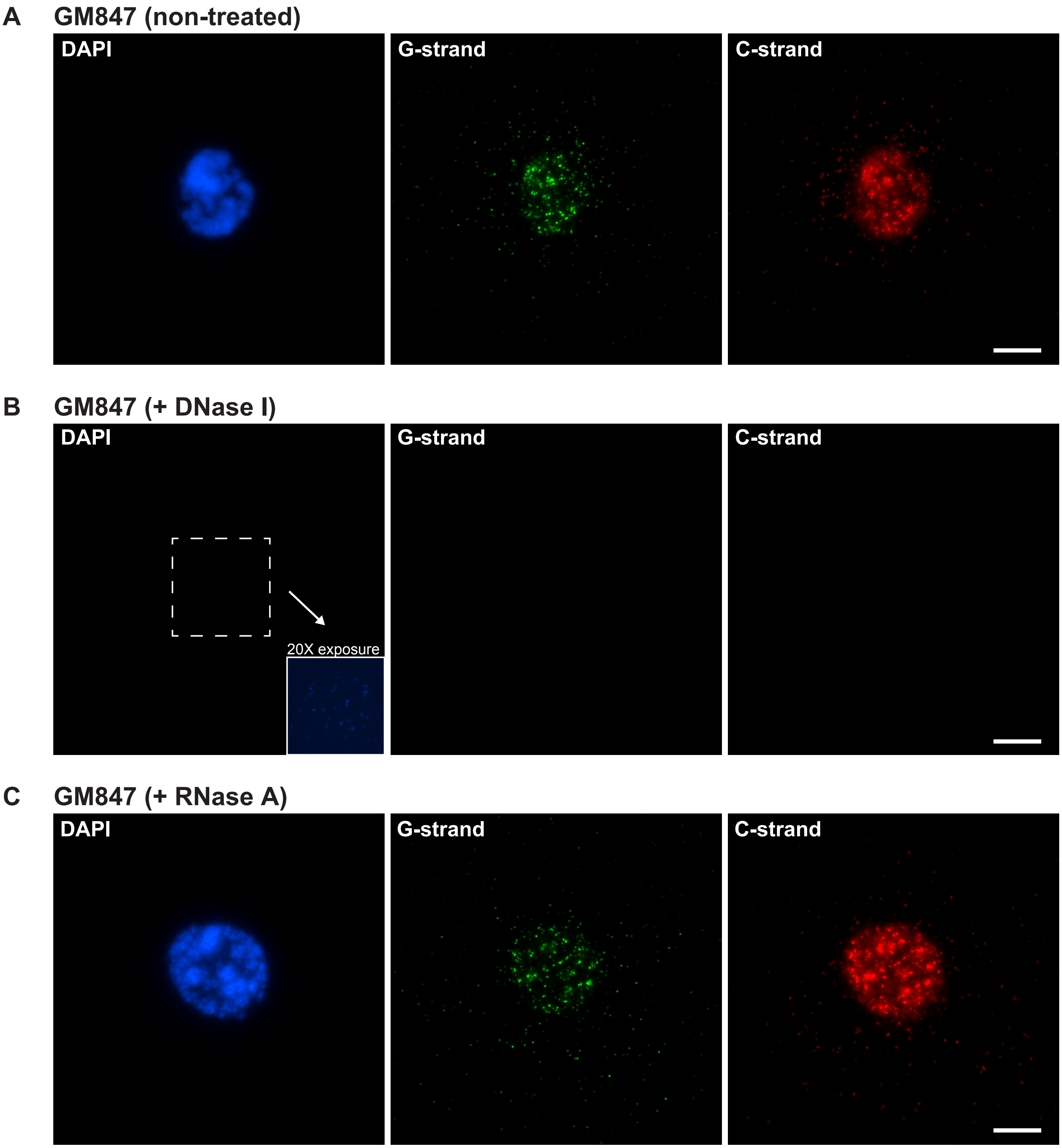

Supplementary Figure S1. Effects of DNase I and RNase A treatment on telomere-repeat fluorescent signals in the Halo-FISH assay. Representative single plane (not deconvolved) images of GM847 ALT cells subjected to the Halo-FISH protocol with (**A**) no treatment, (**B**) treatment with DNase I (100Units/ml) or (**C**) treatment with RNase A (100µg/ml) for 2 hours at 37°C prior to probe hybridization. Cells were PNA probed for G-strand telomere-repeat DNA (green), C-strand telomere-repeat DNA (red) and counterstained with DAPI (blue). For the DNase I-treated nuclei, an embedded DAPI image obtained by 20X over-exposure is shown displaying the undigested DNA remnants of the nuclear core (punctate DAPI staining) that were used to localize cell nuclei in the field of view of the microscope objective. Scale bar, 10µm.

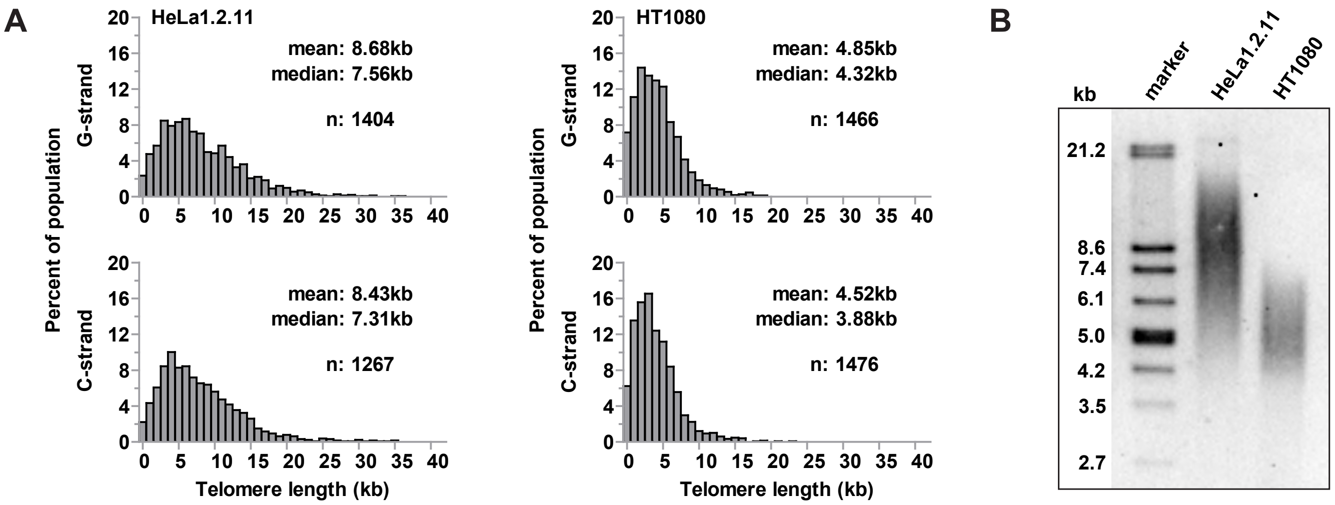

Supplementary Figure S2. Telomere length analysis of HeLa1.2.11 and HT1080 cells. (**A**) The length distributions of telomere-repeat DNA foci localized to the nuclear core of deproteinized HeLa1.2.11 and HT1080 nuclei in the Halo-FISH assay. The mean and median lengths, as well as the number (n) of telomere-repeat DNA foci measured are indicated with each plot. Measurements are obtained from a minimum of 30 cells. (**B**) Southern blot of *Hin*fI/*Rsa*I-digested genomic DNA from HeLa1.2.11 and HT1080 cell lines. The blot was hybridized with a TTAGGG-repeat probe to detect telomere restriction fragments. The average telomere length of HeLa1.2.11 and HT1080 cells is estimated to be 7.77kb and 5.21kb respectively, using the analysis algorithm described in Kimura et al., 2010.

Supplementary Reference:

Kimura,M., Stone,R.C., Hunt,S.C., Skurnick,J., Lu,X., Cao,X., Harley,C.B. and Aviv,A. (2010) Measurement of telomere length by the Southern blot analysis of terminal restriction fragment lengths. *Nat. Protoc.*, **5**, 1596-1607.

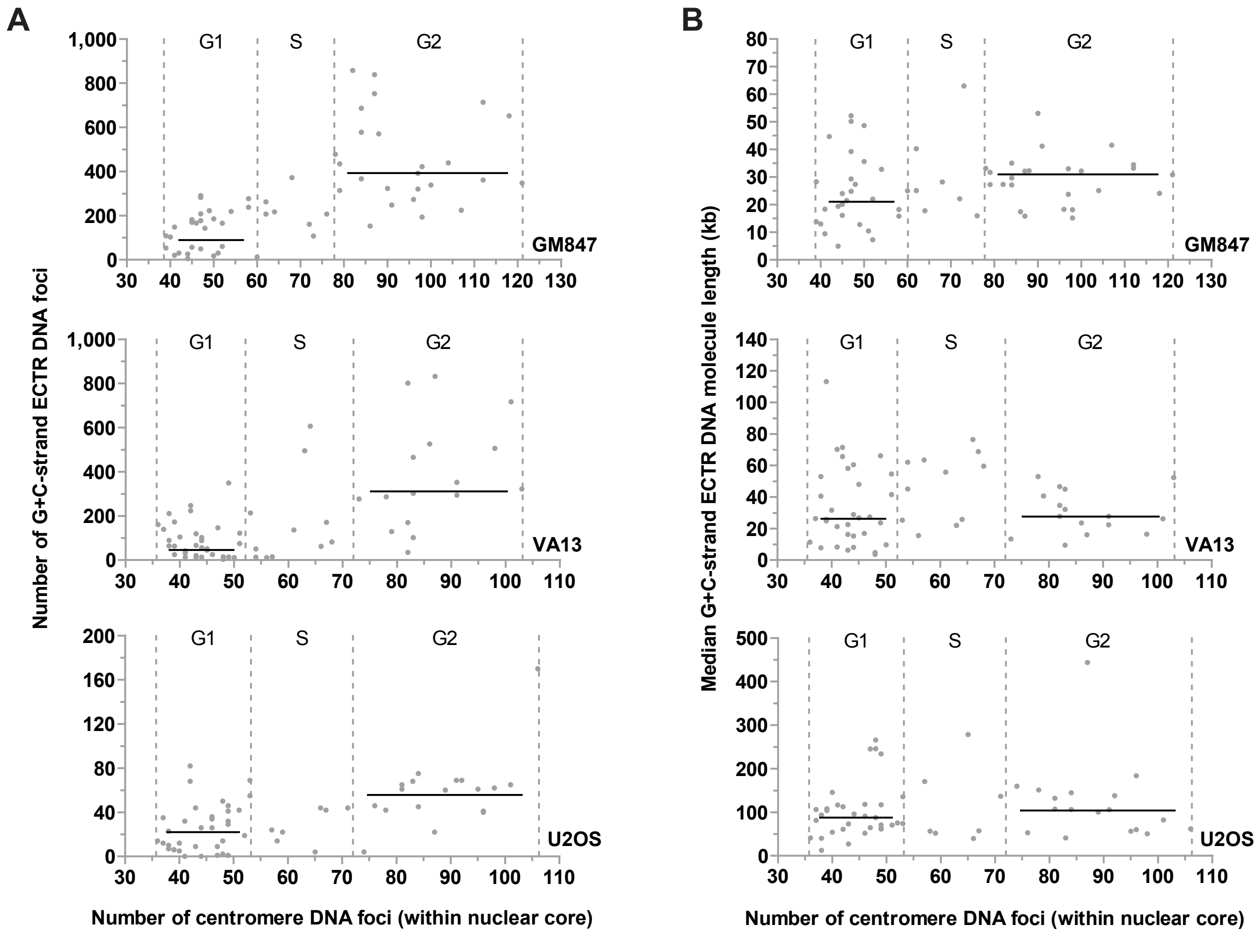

Supplementary Figure S3. Analysis of ECTR DNA and cell cycle progression in human ALT cells. (**A**) Correlation analysis, per nucleus, between the number of G+C-strand ECTR DNA foci and the number of nuclear core-localized centromere DNA foci in GM847, VA13 and U2OS ALT cells. (**B**) Correlation analysis, per nucleus, between the median G+C-strand ECTR DNA molecule length and the number of nuclear core-localized centromere DNA foci in GM847, VA13 and U2OS ALT cells. Cells are classified into G1-, S- and G2-phase enriched fractions through their nuclear core-localized centromere DNA foci counts. The cell nucleus with the fewest nuclear core-localized centromere DNA foci defines the lower G1-phase boundary, while the nucleus with the most defines the upper G2-phase boundary. The lower G2-phase boundary is estimated to be twice the number of centromere DNA foci counted in the lower G1-phase boundary defining nucleus. In a similar fashion, the upper G1-phase boundary is estimated to be half the number of centromere DNA foci counted in the upper G2-phase boundary defining nucleus. Median bars for G1- and G2-enriched populations are displayed.

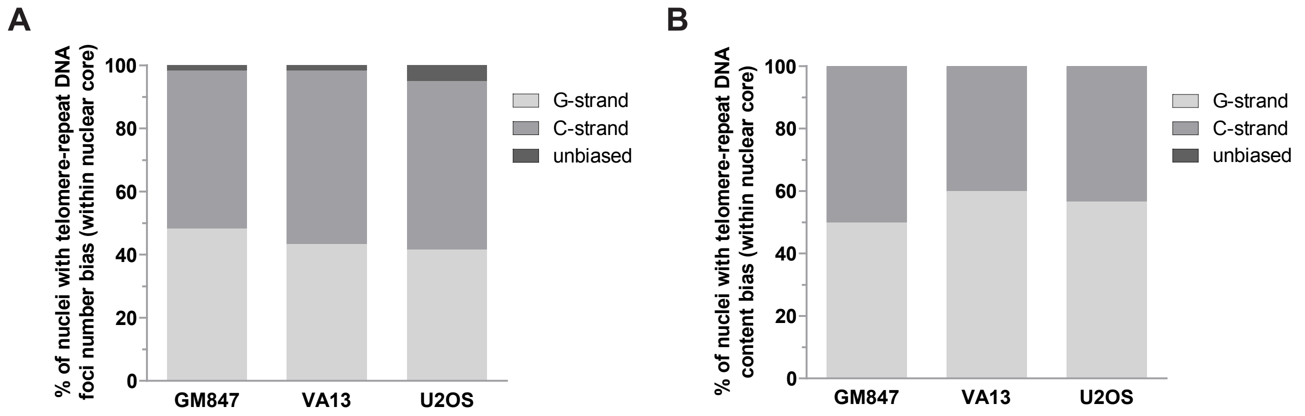

Supplementary Figure S4. Nuclear core telomere-repeat DNA strand biases in human ALT cells. (**A**) The percentage of GM847, VA13 and U2OS ALT cells that show G- or C-strand biases in the number of nuclear core-localized telomere-repeat DNA foci. (**B**) The percentage of GM847, VA13 and U2OS ALT cells that show G- or C-strand biases in nuclear core-localized telomere-repeat DNA content.
